# Polyphenols Content, Antioxidant Activity, and Cytotoxicity Assessment of *Taraxacum officinale* Extracts Prepared through the Micelle-Mediated Extraction Method

**DOI:** 10.3390/molecules24061025

**Published:** 2019-03-14

**Authors:** Michał Miłek, Dana Marcinčáková, Jaroslav Legáth

**Affiliations:** 1 Department of Chemistry and Food Toxicology, Faculty of Biology and Agriculture, University of Rzeszow, Ćwiklińskiej 1a, 35-601 Rzeszów, Poland; mmilek@ur.edu.pl; 2 Department of Pharmacology and Toxicology, University of Veterinary Medicine and Pharmacy in Košice, Komenského 73, 041 81 Košice, Slovakia; jaroslav.legath@uvlf.sk; 3 Department of Biotechnology and Bioinformatics, University of Technology, Powstańców Warszawy 6, 35-959 Rzeszów, Poland

**Keywords:** antioxidants, cytotoxicity, micelle-mediated extraction, xCELLigence system, UHPLC-MS/MS

## Abstract

This experiment was conducted with extracts prepared from dandelion (*Taraxacum officinale* F. H. Wigg) leaves and flowers, using the micelle-mediated extraction method, with the surface active compound Triton X-100 and water–acetone as the extraction solvents. Extracts were, first, examined for the content of total phenols and the antioxidant capacity. All extracts showed good anti-radical properties, especially for leaves, in comparison to the flower samples. Flavonoids (mainly luteolin derivatives) and phenolic acids, predominated among the determined polyphenols. Quantitative analyses indicated acetone extract to be the richest in phenols (up to 0.535 mg/mL), in the case of dandelion leaves, and Triton X-100 extract in the case of flowers (0.385 mg/mL). Extracts were also evaluated for cytotoxicity to the model cell line (epithelial rabbit kidney cells RK13), using the colorimetric 3-[4,5-dimethylthiazole-2-yl]-2,5-diphenyltetrazolium bromide (MTT) test and the real-time cell analysis method ((RTCA); *xCELLigence* system). The obtained results indicated that surfactants, especially non-ionic ones, can be effectively used as modifiers in the aqueous extraction of phenolic compounds from plant materials. An advantage over the traditional organic solvents is their non-flammability. Furthermore, surfactants might also be used at low concentrations. Studies on cell lines, however, indicated the cytotoxic effect of this type of compound, even in the trace amounts present in the extracts.

## 1. Introduction

Despite the existence of many modern, efficient and economically advantageous methods for the extraction of various plant metabolites, these systems of extraction can be further developed, especially through the selection of appropriate solvents. The aim is to create, or improve upon, extraction systems, so that they are effective, inexpensive, environment-friendly, and safe for humans and the environment. There are many reports about the use of surface active compounds in extraction as an alternative to organic solvents [1,2,3]. Micellar-mediated extraction is a promising technique intended to reduce the use of toxic, flammable and often problematic organic solvents. It can be used to obtain or purify various types of compounds, including those of plant origin. Surfactants, as an extraction media, belong to the trends of “green chemistry”, which focuses on the elimination of production and the usage of substances hazardous for the environment and health. Solutions of surface-active compounds used in the extraction in low concentrations are safe, non-toxic, non-flammable, and do not cause problems with the utilization of waste. Furthermore, extracts prepared by this method might potentially have applications in other fields, such as in the food, cosmetic, and pharmaceutical industries, hence, the impact on live organisms must also be evaluated. There are many examples in which such types of extraction techniques have been utilized, to obtain biologically active plant compounds, such as polyphenols [4,5,6,7].

The most commonly used surfactants in the extraction processes of secondary metabolites of plants, include both ionic compounds, such as sodium dodecyl sulphate (SDS) or cetyl trimethylammonium bromide (CTAB) [4,7,8], and non-ionic compounds, such as Tritones (X-100 or X-114), Tweens (20 and 80), and Genapol X-080 [2,5,9,10]. Two characteristic fragments can be distinguished in the structure of these types of compounds—a non-polar “tail” and a polar “head”. This chemical structure contributes to the surface active properties of these compounds. This phenomenon is based on decreasing the surface tension of aqueous solutions, as well as the interfacial tension in the case of systems that utilize immiscible liquids. Another special feature of surfactants is the ability to associate in structures, called micelles. Inside the micelles, various compounds can be solubilized, which is the basis of the micelle-mediated extraction method [3].

Dandelion (*Taraxacum officinale* F. H. Wigg.) is a well-known medicinal plant containing numerous polyphenolic compounds, classified as flavonoids (including luteolin, quercetin, chrysoeriol and their glycosides) and phenolic acids (caffeic, chlorogenic, coumaric, caftaric acid and others). Many beneficial properties are attributed to this plant, including diuretic, choleretic, anti-inflammatory, antioxidative, anti-hyperglycemic, or anticancer action [11].

In our study, the dandelion leaves and flowers were used for the preparation of the extracts. In the micelle-mediated method, non-ionic surfactant Triton X-100 was employed as an aqueous extraction modifier. This compound characterized by a high hydrophilic-lipophilic balance (HLB) coefficient value, is a good solubilizer and it is used as a factor that can improve the recovery of various types of analytes, from a solid matrix. In the second type, of extraction, aqueous acetone solution (30%) was used. Acetone is a polar solvent commonly used for the extraction of plant polyphenols. The extracts prepared with these two different methods were subjected to cytotoxicity tests, using rabbit epithelial kidney cell line (RK13). The metabolic activity of the cells that were exposed to both types of extracts were evaluated by colorimetric assay (MTT test) and cell proliferation was evaluated using the xCELLigence system.

## 2. Results and Discussion

The results of total phenolic content and antioxidant capacity evaluation are summarized in Table 1. The results of the total phenolic content analysis indicated that aqueous acetone is a better extraction solvent than Triton X-100. We extracted more polyphenols from the dandelion leaves than flowers; it could be related to the degree of the plant material dryness and fineness. The flowers contained more water and after pulverizing with mortar and pestle, we obtained the material for extraction, with a smaller degree of fragmentation. This could have an effect on solvent penetration and it consequently resulted in less polyphenol recovery.

Antioxidant properties of all extracts were measured by three methods. The results are in good correlation and generally, a higher polyphenol content is reflected in a higher antioxidant capacity. The decrease in antioxidant capacity for most micellar extracts, in comparison to aqueous acetone, can be explained by the solubilization effect of polyphenols, in surfactant micelles, as well as a limited accessibility of antioxidants to DPPH radical or oxidized metal ions. A similar effect was observed by scientists investigating the micellar extraction of polyphenols from elderberry blossom [12] and in research on the antioxidant effects of rutin and ascorbic acid, in non-ionic surfactant micelles [13]. In spite of this, the results confirmed the high antioxidant potential of the obtained dandelion extracts. Data on such properties can be found in the reports of many authors [14,15,16].

There was a good correlation between the total phenolic content and the antioxidant activity of the tested samples (Table 2), which indicated that polyphenols are the main compounds affecting the antiradical properties of the extracts. Results obtained using different methods were also in good correlation (*r* values above 0.8), the highest stated for DPPH and the reducing power method (*r* = 0.888).

The UHPLC-MS analyses were also carried out in order to obtain information about the qualitative composition of plant extracts and then about the quantitative content of selected polyphenols in the samples. Qualitative analysis data for *T. officinale* leaves are recorded in Table 3 and for the flowers, in Table 4. The qualitative identification of polyphenolic compounds was conducted, based on the literature data and comparison of the retention times and fragmentation patterns with several analytical standards (luteolin, luteolin-7-glycoside, chrysoeriol, and chicoric acid).

The formation of solvent adducts [M + FA − H]^−^ and molecular complexes of polyphenols ([2M − H]^−^) was a factor facilitating the recognition of molecular ions [M − H]^−^. The dominant metabolites in the analyzed extracts were phenolic acids and their esters, mainly chicoric acid and caffeoylquinic acids. Caffeoylquinic acid isomers could be distinguished by their characteristic fragmentation patterns: 3-*O*-caffeoylquinic acid (neochlorogenic acid) forms relatively intense (ca. 50% of base peak) daughter ion at *m*/*z* 179, and either absent or weak daughter ions, in the presence of 1-*O*-caffeoylquinic acid and 5-*O*-caffeoylquinic acid [17]. For the exact distinction of these two isomers, a comparison of the retention times in the chromatographic separation should be done, with use of analytical standards. Similarly, in the case of dicaffeoylquinic acid, for an exact identification, a comparison with a standard would be necessary. Organic solvent was more effective for isolation of the glycoside forms, in comparison to surfactant solutions, probably due to differences in polarity. In addition to the aforementioned luteolin aglycone and cynaroside, we noted the presence of other luteolin glycosides (indicated in Table 4 and Table 5 as I, II, or III) and also luteolin diglucoside. Only one of the luteolin glycosides, identified with a high confidence (by comparing the retention time with the standard analyses), was luteolin-7-glycoside. Moreover, in flower extracts, we identified chrysoeriol, a 3′-methoxy derivative of luteolin. The presence of this compound confirmed previous reports [18,19]. Apigenin, isorhamnetin or their glycosides were not detected, although the presence of these compounds in dandelions have been mentioned elsewhere [11]. Some compounds remained unidentified, e.g., a large peak present in all chromatograms (at approximately 4.9 min), which was characterized by a molecular ion peak of *m*/*z* 469. All polyphenols were eluted from the chromatographic column in less than 10 min, beyond that, only peaks from the solvents (surfactants) were visible.

Four polyphenols—luteolin, luteolin-7-glycoside (cynaroside), chrysoeriol and chicoric acid, were selected for the quantitative analysis in the extract samples. Quantitation was conducted in a multiple reaction monitoring (MRM) mode, using electrospray ionization (ESI) source parameters optimized for standards. Data on MRM pairs used for calculations, collision energy values, and validation parameters of quantitative methods are summarized in Table 5.

Determined content of the four analyzed polyphenols, expressed in mg/mL of the extract is recorded in Table 6.

Luteolin was extracted with a better yield from the acetone extracts of dandelion leaves than the Triton X-100 extracts. In reverse, the Triton X-100 flowers extracts produced a greater yield. The better solvent for cynaroside extraction was acetone (in the dandelion leaves extract). Chrysoeriol presented only in the *T. officinale* flowers, was detected in significantly higher amounts, after the acetone extraction. Interestingly, chicoric acid was extracted only with the use of acetone–water mixture. In the micellar-assisted extracts, it occurred in amounts below the limit of quantitation. A relatively high value of detection and quantitation limit for this compound can also be important.

**Figure 1:**
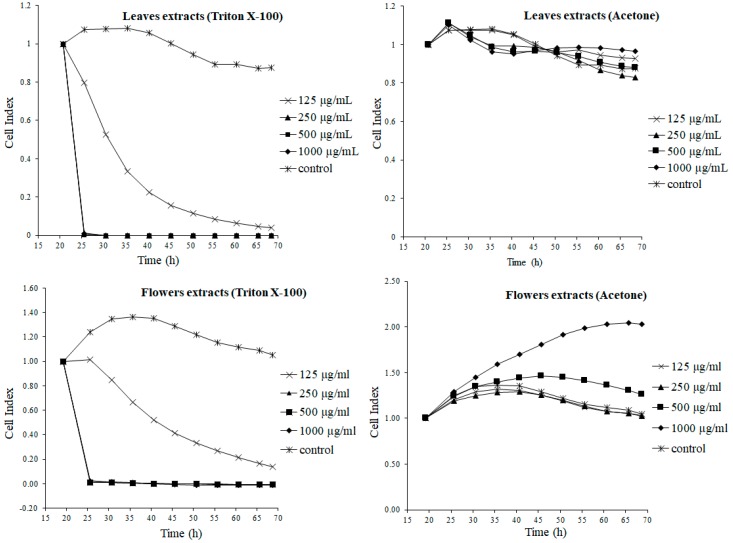
Real-time monitoring of dynamic cell changes, after 48 expositions to flowers and leaves extracts, prepared with the Triton X-100 and acetone.

To evaluate the impact of the extracts prepared by the different extraction systems, on a live organism, the rabbit kidney cell line RK13 was used. We evaluated the changes of the cells in real time, during the whole time of exposure to dandelion extracts, by using a real-time cell analyzer. This *xCELLigence* system allows the monitoring of the status of cells, their behavior (adherence, proliferation and change in morphology) in real-time. All these changes were expressed as a cell index (CI). Figure 1 shows the effect of extracts that were prepared with the surfactant Triton X-100 and aqueous acetone. The surfactant have shown a toxic effect, shortly after the addition to cells. The presence of surfactants in the tested extracts resulted in their cytotoxicity to the cells of the RK13 line. A significant decline in CI (*p* < 0.001) was recorded after cell treatment with all tested concentrations, except the surfactant extract of flowers, at the lowest concentration—125 µg/mL—where the CI increased significantly (*p* < 0.001), in comparison to the control cells without treatment. The cytotoxic effect prevailed over the expected nephroprotective effect resulting from the content in polyphenol extracts, mainly flavonoids. It can be explained by the dissolution of the phenolic compounds in the surfactant micelles, which can significantly impede the penetration into cells [3].

On the contrary, the CI of the cells treated with water–acetone dandelion leaves extract remained at a similar level to the control sample, during the whole treatment period. Within a short time, after the introduction of the extracts, there was a slight increase in the CI value, for all tested samples. In the further course, the CI curves slightly decreased and stabilized until the end of the experiment. These results prove a lack of the cytotoxic activity of the tested aqueous acetone extract. Similar to the dandelion leaf micellar extracts, the micellar flower extract also showed a strong cytotoxic effect (*p* < 0.001). Only the less concentrated sample (125 µg/mL) did not cause a swift decrease to zero, in the cell index value. After the slight initial CI increase, in the further course of the experiment, there was a continuous decline, but it did not reach zero, during the time of monitoring.

Completely different results were recorded in the cells treated with water–acetone flower extract. A continuous increase in the CI, significantly higher in comparison to the control, is evident at the highest tested concentration (1000 µg/mL), the value of which stabilizes at about 40 h into the treatment, but was at a much higher level, still, than the control sample (*p* < 0.001). A significant growth in the CI value was also observed in the cells treated with 500 μg/mL of the flower acetone extract (*p* < 0.001), with respect to the control. The increase in the CI indicates the positive impact of the extract on cell proliferation. The course of the other curves (125 and 250 µg/mL) was consistent with the course of the control curve. It meant that no negative impact on cell behavior was recorded.

The second method for cytotoxicity evaluation employed in our research was an MTT test. This test is based on measurement of metabolic activity of the mitochondria, within the cells exposed to the tested substance. The mitochondrial enzyme succinate dehydrogenase catalyzes the reaction in which colorful formazans are created. Spectrophotometrical measurement of the formed formazan allows one to evaluate the metabolic activity of the cells and the cytotoxic effect of the tested substance [20]. The results of the MTT test, carried out on RK13 cells, after 48 h of exposure to the tested extracts, are shown in Table 7, which summarizes the values obtained for both in vitro methods used for the cytotoxicity testing.

In contrast to the extracts prepared with micellar support, those prepared using organic solvent (aqueous acetone) were less toxic for cells. Moreover, at lower concentrations (125–500 µg/mL) of the leaves extract, an increase in the metabolic activity was recorded, in comparison to the control (*p* < 0.001). For the dandelion flower extract, the value increased, which would be indicative of the action supporting the metabolism of the kidney cells, by the components comprised within the extract. The metabolic activity of the cells decreased and the cytotoxic effect appeared during the treatment with flower extract. The surfactant did not evaporate completely, during the lyophilization of the raw extracts. The remaining surfactant acted on the cells after the dry extracts were dissolved in water. The MTT test results clearly indicated the cytotoxic effect of the extracts prepared with Triton X-100. We can assume that these compound residues were responsible for the cytotoxic effect. With the increase of the tested concentration, the metabolic activity dropped to near zero. Comparison of the cytotoxicity, depending on the concentration of the tested extract, led to the conclusion that the toxicity of the extracts for the cells increases with increasing concentration.

The toxicity of the dandelion, using the HepG2 human hepatocellular cell line and the MTT test, was studied by Koo et al. [21]. Although the authors used a different type of extraction solvent and a cancer cell line, they showed that the preparations from this plant induced cell apoptosis by stimulating the production of TNF-α tumor necrosis factor and IL-1α interleukin. The influence of another dandelion species extracts (*Taraxacum hispanicum*) on the same cell line (HepG2) was investigated by Laranjeiera et al. [22]. The ethanolic extract of *T. hispanicum* caused an increase in the metabolic activity of the cells, between 24 and 48 h of the treatment (especially at lower concentrations), which suggests a hepatoprotective effect of dandelion, in moderate doses. The observed effect depended on the concentration of the extract. The protective effect of the dandelion, also in combination with milk thistle (*Sylibum marianum*), in kidneys exposed to carbon tetrachloride (CCl_4_) has been demonstrated by in vivo studies carried out by Karakuş et al. [23]. It can be assumed that a water–acetone extract of dandelion leaves has a nephroprotective effect. The nephroprotective effect of this plant has been confirmed [23]; it is also one of the traditional herbs commonly used in kidney diseases, acting as a diuretic [24,25] and a natriuretic [24,26]. By increasing urine production and preventing hyperkalemia, dandelion could be one of the factors that protect against kidney disease.

To obtain biologically active compounds from plants, it is necessary to optimize the extraction method and a suitable extraction solvent must be used. Acetone as an organic solvent is toxic and flammable and might form products which are dangerous for the environment [27], but it is very effective in the extraction of phenols with a high molecular weight, as well [28]. The industry is in continuous search for new alternatives, e.g., from the group of surfactants (Triton X-100). Surfactants decrease the surface tension of aqueous solutions and assemble into the micelles prepared for the micellar-mediated extraction of polyphenols [7].

The main objective of our research was to evaluate and compare the biological properties of dandelion extract, prepared using two different extraction solvents—commonly used organic solvent acetone and non-ionic detergent Triton X-100.

In evaluation of the antioxidant capacity and the total phenol content, we can assume that acetone was a better extraction solvent, when compared to Triton X-100. In the micellar extraction, the polyphenols were solubilized in surfactant micelles, so their availability was limited. Our findings are not consistent with results obtained by [7]. The authors claimed that the surfactant water solution had the highest extraction efficiency. This confirmed that various extraction solvents could be used for extraction of polyphenols with different chemical structures.

Triton X-100 and other non-ionic surfactants are also used in the pharmaceutical and cosmetic industry. Therefore, it is necessary to study the cytotoxic effects of surfactants on live organisms. It was confirmed that cytotoxicity increases with a growing concentration of Triton X-100 [29,30,31,32]. In our experiment, only the lowest concentration of the Triton X-100 extract did not cause cell death. The higher concentration of extracts caused a drop in proliferation, to zero, shortly after the cell exposure. The surfactant had not evaporated completely, during the lyophilization process, and some residues remained in the extract. We can assume that cytotoxicity of the surfactant depends on the concentration, even in dry lyophilized extracts.

Different classes of surfactants were also tested, also on cell lines, e.g., on human fibroblasts. Determination of LC50 values enabled the classification of the compounds’ toxicity. The highest LC50 value was determined for Tween 80, which led to the conclusion that among the tested surfactants, it was the least toxic to fibroblasts [30]. The Triton X-100 used in this study was placed in the cited studies, at the forefront of a series of cytotoxicity. Although the authors clearly did not provide the mechanism for the toxic action of surfactants, it could be supposed that they can affect the integrity of cell membranes and reduce the adherence of cells. A better choice for extraction would be to use another modifier, which is not considered to be highly toxic, such as polyethylene glycol (PEG 400) and polypropylene glycol, which, in the research of Hamzeloo-Moghadam et al., were considered to be non-toxic, similar to methanol or ethanol [33].

Many studies have showed that the action of polyphenols depends on their concentration, and at low concentrations, they might promote cell proliferation. Regarding cancer cells, the desired effect is inhibition of their growth and induction of apoptosis, while the protective effect for healthy cells meant promoting their cell division and differentiation. The influence of the polyphenol-rich (especially anthocyanins) extracts from blueberries (*Vaccinium angustifolium*), on the proliferation of liver cancer cells (HepG2 line) is described by Shafiee-Kermani et al. [34]. In contrast to the high concentrations of the extract used in the studies on cancer cell lines, low doses (within 25 μg/mL) caused an increase in HepG2 cell proliferation. It was shown that the increase was independent of the oxidative status of the cells [34]. A similar concentration and effect relationship at the cellular level was also observed for the polyphenol extract of the evening primrose extract (*Oenothera paradoxa*). This plant contains various polyphenols, including flavonoids, proanthocyanidins, and phenolic acids, similar to those found in *Taraxacum officinale* (caffeoylquinic acids). The low concentration of this extract stimulated the proliferation of Caco-2 cells (human colon adenoma), after 24 and 48 h of in vitro testing. However, no effect on cells at the higher concentrations of the extract was observed [35].

## 3. Materials and Methods

### 3.1. Plant Material and Extraction

Common dandelion (*Taraxacum officinale* F.H. Wigg) leaves and flowers were collected from a natural habitat (Rzeszów, south-west Poland, 50°02′N 21°53′E) in May 2016. After botanical identification, the plant material of several plants was dried at a temperature below 30 °C, without exposure to sunlight. A voucher specimen (TO 3/5/16) of the plant was deposited in the Department archive. Dried raw material was pulverized with mortar and pestle. Pulverized plant material in amount 1.5 g was placed in a screw-cap 50 mL centrifuge tube and mixed with 20 mL of the extraction solvent. Aqueous solution of Triton X-100 (Sigma Aldrich; St. Louis, MO, USA) was used for micellar extraction of the dandelion leaves, at a concentration of 2% (*v*/*v*). Aqueous acetone (Chempur, Tarnowskie Góry, Poland) at a concentration of 30% was applied as a control of the organic solvent. Extraction was carried out in an ultrasonic bath (Sonorex RK 31, Bandelin, Berlin, Germany). After ultrasonic extraction for 30 min, the mixture was centrifuged at 6500× *g* for 10 min. The supernatant was collected and the extracts were freeze-dried and stored at −20 °C, until the testing. Extracts were diluted with water, shortly before the experiment, to the final concentrations of 1000 µg/mL and to 125; 250; 500; 1000 µg/mL for in vitro cytotoxicity evaluation.

### 3.2. Determination of Total Phenolic Content

*Taraxacum officinale* extracts were analyzed for the total phenolic content by the Folin–Ciocalteu method [36]. The reaction mixture was prepared by mixing 0.1 mL of crude extract and 0.2 mL of the Folin–Ciocalteu reagent (POCH, Gliwice, Poland) dissolved in water (1:10). After 5 min of equilibration, 0.8 mL of 700 mM sodium bicarbonate solution (Chempur, Tarnowskie Góry, Poland) was added and well-shaken. After incubation for 1 h, at room temperature, in darkness, the absorbance of the mixture was read at 765 nm, against a reagent blank, using a UV-Vis spectrophotometer (UV-160A, Shimadzu, Kyoto, Japan). The total phenolic content was expressed as (+)-catechin equivalents (mg CE/g DW of extract), based on a prepared standard curve. The samples were prepared in triplicates and the mean value was calculated.

### 3.3. Antioxidant Assays

#### 3.3.1. DPPH Radical Reduction Measurement

DPPH (2,2-diphenyl-1-picrylhydrazyl) radical reduction was measured, according to Brand–Williams et al. [37], with slight modifications. The sample of crude extract (0.1 mL) was rapidly mixed with 3.9 mL of DPPH (Sigma Aldrich; St. Louis, MO, USA) methanolic solution (0.1 mM). After 15 min of incubation in dark, the absorbance of the mixture was measured at 515 nm, against the blank (methanol). The reduction of DPPH radical was calculated, according to Equation (1):

(1) $$\%\mathrm{DPPH}\;\mathrm{reduction}=\;\frac{\left(A_{0}-{\;A}_{15}\right)}{A_{0}}\;\times 100$$

where A_0_ is the absorbance of pure DPPH, without the extract; A_15_ is the absorbance of the mixture after 15 min of incubation. The results were converted to the trolox equivalents (mg TE/g DW of extract), based on a prepared standard curve of trolox. The samples were prepared in triplicates and the mean value was calculated.

#### 3.3.2. Reducing Power Measurement

The reducing power (Fe^3+^ → Fe^2+^) was evaluated, according to the method of Oyaizu [38], with slight modifications. Crude plant extracts (0.5 mL) were mixed with 1 mL of phosphate buffer (0.2 M, pH 6.6) and 1 mL of 1% potassium ferricyanide (POCH, Gliwice, Poland). Then, the reaction mixtures were incubated at 50 °C, for 20 min. After incubation, 1 mL of 10% trichloroacetic acid (POCH, Gliwice, Poland) was added, and the mixtures were centrifuged at 1000× *g* and the upper layers were collected. Subsequently, 0.1 mL of 0.01% ferric chloride (Roth, Karlsruhe, Germany) was added and the samples were diluted with distilled water (1.5 mL). The absorbance was read at 700 nm and the results were converted to ascorbic acid equivalents (mg AAE/g DW of extract), based on a prepared standard curve.

#### 3.3.3. Cupric Ion Reducing Antioxidant Capacity (CUPRAC)

The cupric ion reducing capacity was determined, according to Apak et al. [39]. To a test tube containing 0.02 mL of the extract sample, 0.125 mL of copper(II) chloride (10 mM, POCH, Gliwice, Poland), 0.125 of the neocuproine ethanolic solution (7.5 mM, Roth, Karlsruhe, Germany), and 0.125 mL ammonium acetate (1 M, Chempur, Tarnowskie Góry, Poland) were added and diluted with distilled water (0.605 mL), to obtain a total volume of 1 mL. After 30 min of incubation at room temperature, the absorbance was measured at 450 nm, against a reagent blank. The results were expressed as trolox equivalents (mg TE/g DW of extract), based on a prepared standard curve. The samples were prepared in triplicates and the mean value was calculated.

### 3.4. Chromatographic Analyses (UHPLC-MS)

The UHPLC-MS analyses were carried out using Nexera 2 UHPLC chromatograph, equipped with LC-30AD high pressure pump, degasser, SIL-30AC autosampler, CTO-30A thermostat, and CBM 20A controller unit, coupled with QTRAP 4500 triple quadrupole mass spectrometer (AB Sciex, Framingham, MA, USA), as a detection system (Shimadzu, Kyoto, Japan). The separation was performed using a Kinetex XB-C18 (Phenomenex, Torrance, CA, USA) chromatographic column (50 × 2.1 mm i.d., 1.7 μm particle size), with a precolumn, operated at 25 °C temperature. The mobile phase consisted of 0.05% formic acid in MS-grade water (POCH, Gliwice, Poland) and acetonitrile (POCH, Gliwice, Poland). The gradient elution program was—10% to 100% of acetonitrile (0–20 min) and 100% acetonitrile (20–25 min). The injection volume of samples was 2 μL, flow rate—0.4 mL/min. As an ionization method, electrospray was used (ESI). Negative ion mass spectra were recorded using the Analyst 1.6.2 software (AB Sciex, Framingham, MA, USA), in the range *m*/*z* 100–1000 (in the qualitative analysis experiment).

Selected polyphenols (luteolin, luteolin-7-glucoside, chrysoeriol, quercetin-3-glucoside, rutin and chicoric acid; Roth, Karlsruhe, Germany) were analyzed, quantitatively, with MRM (multiple reaction monitoring) mode. The MRM conditions were determined and optimized using phenolic standards, and the polyphenols were quantified in extracts, with the use of standard curves obtained for commercial standards. Results were expressed as milligrams of the compound per 1 g of extract dry weight (DW).

### 3.5. Cytotoxicity Tests

#### 3.5.1. Cell Cultivation and Treatment

Epithelial RK13 cells (rabbit kidney epithelial cell line) were obtained from the American Type Culture Collection (ATCC^®^CCL-37TM). Cells were cultivated in the Earl’s Minimal Essential Medium (EMEM; Lonza, Valais, Switzerland), supplemented with 10% (*v*/*v*) fetal bovine serum (FBS; Lonza, Valais, Switzerland) and 50 mg/L gentamicin (Sigma Aldrich; St. Louis, USA), in a humidified atmosphere of 5% CO_2_, at 37 °C. In the experiments, RK13 cells were cultivated in a complete cultivation medium, without antibiotics, and were regularly checked for absence of mycoplasma contamination [40].

The lyophilized extracts were dissolved in sterile water, to obtain concentrations of 125, 250, 500, and 1000 μg/mL, shortly before the experiment, and were added to the adherent cells, after 24 h of cultivation in the EMEM medium (37 °C, 5% CO_2_).

#### 3.5.2. xCELLigence Assay

The real-time monitoring of cell response to the extracts’ exposure was monitored by using the xCELLigence system or the real-time cell analyzer (RTCA; Acea Biosciences Inc., San Diego, CA, USA). This system has already been described by many other authors [41,42]. It utilizes a series of micro wells, the bottoms of which are covered at 80%, with microelectrodes that measure the cell viability, by monitoring cell proliferation and morphology, using a dimensionless unit called the cell index (CI), which is based on the impedance changes caused by cells interacting with the microelectrodes. Analysis of cell proliferation was performed, according to the manufacturer’s instructions [43].

At first, 100 μL of the cell medium (EMEM culture medium) was added into the 16-well plates, covered with gold microelectrodes (E-plates, Roche, Applied Science, Mannheim, Germany), for the background measurements. Then, the RK13 cells were seeded in the plate (1.5 × 10^4^ cells/well in 50 μL of culture medium. The plates were placed in an incubator, at 37 °C, in 5% CO_2_ atmosphere. After 20 h of incubation (cells within a log phase), the tested extracts dissolved in sterile water were added to the cells, at final concentrations of 125, 250, 500, and 1000 μg/mL. The cells were incubated for 48 h and the cell index was measured, automatically, by the RTCA system, once per hour, until the end of the experiment). Cells not exposed to the tested extracts served as the control.

#### 3.5.3. MTT Test

The cells were seeded in a 96-well plate (Greiner-bio-one, Kremsmünster, Austria), at a concentration of 2.2 × 10^4^ cells/well in 100 μL, and after 24 h of cultivation, in an incubator treated with the extracts, for the next 48 h. After 48 h of exposure, 3-[4,5-dimethylthiazole-2-yl]-2,5-diphenyltetrazolium bromide (MTT) test was performed [20]. The culture medium was removed and replaced by a fresh medium with MTT (0.5 g/L). After an additional incubation for 4 h in the dark, the concentrated dimethylsuphoxide was added to the wells in, order to properly dissolve the formed formazan. Finally, Sorensen’s glycine buffer (0.1 mol/L glycine, 0.1 mol/L NaCl, pH 10.5) was used to stabilize the final reaction product. Absorbance of the samples was measured, using a microplate reader (Synergy HT; Biotek, Winooski, VT, USA), at 570 nm, against a blank. Metabolic activity (% MA) of the cells, was calculated, in accordance with Equation (2):

(2) $$\%\mathrm{MA}=\;\frac{A_{\mathrm{extract}}}{A_{\mathrm{control}}}\;\times 100$$

where A_extract_ is the absorbance of the sample exposed to the extract and A_control_ is the absorbance of the control sample (cells without extract exposition, 100% metabolic activity). The results were expressed as mean value (*n* = 3), and standard deviation was calculated.

### 3.6. Statistical Analyses

Results were expressed as means ± standard deviation (SD; *n* = 3). The data was evaluated using the GraphPad Prism version 3.00 software (GraphPad Software, San Diego, CA, USA) by one-way analysis of variance (ANOVA), followed by Dunnett’s multiple comparison test. The correlations among the total phenols content and the antioxidant capacity data, were analyzed, through the Pearson *r* coefficient. The significant differences in the level of the total phenols, antioxidant capacities and quantitative content of particular phenols, were calculated through a one-way analysis of variance, followed by Tukey’s honest significant difference test (*p* < 0.05), using the Statistica 13.1 software (StatSoft, Tulsa, OK, USA).

## 4. Conclusions

The extracts of the common dandelion leaves and flowers, made with different solvent systems (aqueous acetone and Triton X-100 solution), were analyzed for their antioxidant properties, polyphenols content, and the effect on live organisms. In terms of the total content of phenolic compounds, determined by the Folin–Ciocalteu method and expressed in mg equivalents of (+)-catechin per 1 mL of the crude extract, extracts made with water–organic mixtures were a bit richer than those derived from micellar extraction. The research also confirmed the significant antioxidant potential of extracts from the studied plants, regardless of the extraction media used. By using various types of solvent systems, it has been shown that it is possible to choose such an extraction medium that allows one to obtain the desired composition and properties of the plant extract. The cytotoxicity testing of selected dandelion extracts, carried out with the two methods, suggests that, even a small amount of the remaining surface active compound in the dry crude extract, might have a toxic effect at the cellular level. However, it must be considered that the commonly regarded non-toxic surfactants might have a negative effect at the cellular level. This effect should be taken into consideration, during the experiment on live organisms, where this group of compounds is used as additives to the extractive media or as ingredients facilitating the dissolution of substances tested on the cell lines. Cytotoxic effects might also be desirable when the subject of research is the cancer cell line. The ability to introduce cells into the apoptosis pathway mighty be beneficial in this situation, due to the significant impact on growth by the anti-cancer properties of test substances, including the plant extracts.

## Figures and Tables

**Table 1:** Total phenolic content and antioxidant capacity of *Taraxacum officinale* extracts.

Extract (Solvent)	TPC (mg CE/g DW)	Antioxidant Capacity		
		DPPH Reduction (mg TE/g DW)	Reducing Power (mg AAE/g DW)	CUPRAC (mg TE/g DW)
Leaves (Triton X-100)	0.410 ± 0.009 ^b^	0.962 ± 0.004 ^b^	1.167 ± 0.078 ^a^	1.165 ± 0.237 ^b^
Leaves (Acetone)	0.535 ± 0.033 ^c^	0.950 ± 0.002 ^b^	1.132 ± 0.012 ^a^	1.908 ± 0.049 ^c^
Flowers (Triton X-100)	0.229 ± 0.010 ^a^	0.294 ± 0.012 ^c^	0.871 ± 0.015 ^b^	0.459 ± 0.069 ^a^
Flowers (Acetone)	0.385 ± 0.008 ^ab^	0.892 ± 0.005 ^a^	0.997 ± 0.016 ^b^	1.041 ± 0.046 ^b^

TPC—total phenolic content; CE—catechin equivalents; DPPH—2,2-diphenyl-1-picrylhydrazyl; AAE–ascorbic acid equivalents; TE—trolox equivalents, DW—dry weight. Data as mean value ± standard deviation (SD; *n* = 3). Means sharing the same superscript letter (in a column) are not significantly different (Tukey’s honest significant difference test, *p* < 0.05).

**Table 2:** Correlation matrix for total phenols and antioxidant capacity assays.

	TPC	DPPH	Reducing Power	CUPRAC
TPC	1.000			
DPPH	0.873	1.000		
Reducing power	0.848	0.888	1.000	
CUPRAC	0.988	0.791	0.798	1.000

TPC—Total Phenolic Content; DPPH—2,2-diphenyl-1-picrylhydrazyl; CUPRAC—Cupric Reducing Antioxidant Capacity.

**Table 3:** UHPLC-MS identification of main polyphenol compounds in *Taraxacum officinale* leaves extracts.

Compound	Retention Time Range (min)	M_w_ (g/mol)	[M − H]^−^ *m*/*z*	MS^2^ Fragment Ions *m*/*z*	Comments
Caffeoyl diglucoside	1.30	504.441	503	341; 311; 179	Only acetone extract
1-*O*-caffeoylquinic acid or 5-*O*-caffeoylquinic acid	1.81–1.84	354.311	353	191; 179	-
3-*O*-caffeoylquinic acid	2.07–2.10	354.311	353	191; 179	-
Caffeoyl glucoside	2.16	342.300	341	311; 191; 179	-
Caffeoylmalic acid	3.07–3.11	296.231	295	179; 133	-
Luteolin diglycoside	3.77	610.521	609	447; 285; 175	Only acetone extract
Chicoric acid	4.21–4.24	474.380	473	311; 293; 179; 135	-
Luteolin-7-glycoside	4.40–4.47	448.380	447	285; 175; 133	-
Unknown	4.88–4.91	-	469	261; 217; 175; 113	-
Dicaffeoylquinic acid	4.99–5.02	516.455	515	353; 191; 179	Probably 1,5-di-*O*-caffeoylquinic acid
Luteolin glycoside II	5.15–5.28	448.380	447	285	-
Luteolin	5.99–6.02	286.239	285	217; 175; 151; 133	-

**Table 4:** UHPLC-MS identification of main polyphenol compounds in *Taraxacum officinale* flowers extracts.

Compound	Retention Time Range (min)	M_w_ (g/mol)	[M − H]^−^ *m*/*z*	MS^2^ Fragment Ions *m*/*z*	Comments
Caffeoyl diglucoside	1.32	504.441	503	341; 311; 179	Only acetone extract
1-*O*-caffeoylquinic acid or 5-*O*-caffeoylquinic acid	1.78–1.82	354.311	353	190; 179	-
3-*O*-caffeoylquinic acid	2.32–2.35	354.311	353	190; 179	-
Luteolin glycoside I	3.52	448.380	447	327; 285; 175	Only acetone extract
Luteolin diglycoside	3.81	610.521	609	447; 285; 175	Only acetone extract
Chicoric acid	4.21–4.26	474.380	473	311; 293; 179; 135	-
Luteolin-7-glycoside	4.40–4.47	448.380	447	285; 175; 133	-
Unknown	4.88–4.91	-	469	261; 217; 175; 113	-
Dicaffeoylquinic acid	4.99–5.02	516.455	515	353; 191; 179	Probably 1,5-di-*O*-caffeoylquinic acid
Luteolin glycoside III	5.15–5.28	448.380	447	285	-
Luteolin	5.99–6.02	286.239	285	217; 175; 151; 133	-
Chrysoeriol	6.90–7.00	300.266	299	284; 256	-

**Table 5:** Quantitative methods data.

Analyte	MRM Pair (Quantifier Ion, *m*/*z*)	Collision Energy (eV)	Standard Curve Equation	Linearity (R^2^)	LOD (ng/mL)	LOQ (ng/mL)
Luteolin	285 → 133	−40	y = 427x − 731	0.9996	3	8
Luteolin-7-glycoside	447 → 285	−34	y = 301x + 1.45 × 10^3^	0.9998	1	2
Chrysoeriol	299 → 284	−28	y = 568x + 3.23 × 10^4^	0.9999	2.5	5
Chicoric acid	473 → 311	−16	y = 12.1x + 4.24 × 10^3^	0.9996	10	100

MRM–multiple reaction monitoring; LOD–limit of detection; LOQ–limit of quantitation.

**Table 6:** Results of the phenols quantification in the *Taraxacum officinale* extracts.

Extract (Solvent)	Luteolin (mg/g DW)	Cynaroside (mg/g DW)	Chrysoeriol (mg/g DW)	Chicoric Acid (mg/g DW)
Leaves (Triton X-100)	1.357 ± 0.136 ^a^	0.007 ± 0.002 ^a^	Not detected	<LOQ
Leaves (Acetone)	5.057 ± 0.490 ^b^	4.787 ± 0.343 ^b^	Not detected	67.867 ± 17.826 ^a^
Flowers (Triton X-100)	22.466 ± 2.854 ^c^	0.446 ± 0.148 ^c^	2.660 ± 0.367 ^a^	<LOQ
Flowers (Acetone)	11.867 ± 1.185 ^d^	0.474 ± 0.056 ^c^	11.756 ± 1.520 ^b^	20.433 ± 1.904 ^b^

Data as mean value ± standard deviation (SD; *n* = 3). DW—dry weight. Means sharing the same superscript letter (in a column) were not significantly different (Tukey’s honest significant difference test, *p* < 0.05).

**Table 7:** The effect of the extracts on the adherence and metabolic activity of the RK13 cells after 48 h exposure. Results are expressed as mean ± SD (*n* = 3).

		Concentration of Extracts (µg/mL)				
		125 µg/mL	250 µg/mL	500 µg/mL	1000 µg/mL	Control
**Leaves (Triton X-100)**						
RTCA	CI	0.24 ± 0.02 ***	0.01 ± 0.01 ***	0.01 ± 0 ***	0.01 ± 0 ***	0.99 ± 0.05
	A (%)	24.41 ± 1.86	0.61 ± 0.2	1.01 ± 0	1.01 ± 0	100 ± 4.73
MTT	OD (nm)	0.05 ± 0.02 ***	0 ***	0 ***	0 ***	0.5 ± 0.06
	MA (%)	1.91 ± 0.06	0.25 ± 0.02	0.56 ± 0.01	0.5 ± 0.04	100
**Leaves (Acetone)**						
RTCA	CI	0.99 ± 0.05	0.95 ± 0.01	0.96 ± 0.05	0.99 ± 0.02	0.99 ± 0.05
	A (%)	100.99 ± 4.76	96.55 ± 1.0	99.37 ± 2.52	100.13 ± 2.02	100 ± 4.73
MTT	OD (nm)	0.71 ± 0.01 ***	0.64 ± 0.04 ***	0.59 ± 0.04 **	0.41 ± 0.02 ***	0.5 ± 0.06
	MA (%)	141.11 ± 3.28	128.12 ± 7.31	117.22 ± 8.64	81.4 ± 4.96	100
**Flowers (Triton X-100)**						
RTCA	CI	0.46 ± 0.11 ***	0.0 ± 0.1 ***	0.0 ± 0.0 ***	0.0 ± 0.0 ***	1.22 ± 0.06
	A (%)	37.43 ± 9.4	0.19 ± 0.39	0.0 ± 0.5	0.17 ± 0.41	100 ± 4.53
MTT	OD (nm)	0.13 ± 0.01 ***	0.01 ± 0.01 ***	0.01 ± 0 ***	0.01 ± 0.01 ***	0.78 ± 0.13
	MA (%)	13.3 ± 1.71	1.08 ± 0.08	1.12 ± 0.06	1.04 ± 0.58	100
**Flowers (Acetone)**						
RTCA	CI	1.18 ± 0.19	1.17 ± 0.22	1.37 ± 0.28 ***	1.78 ± 0.42 ***	1.22 ± 0.06
	A (%)	96.94 ± 15.78	96.11 ± 18.05	111.95 ± 23.02	146.08 ± 34.02	100 ± 4.53
MTT	OD (nm)	0.63 ± 0.05 ***	0.66 ± 0.01 ***	0.63 ± 0.02 ***	0.62 ± 0.03 ***	0.78 ± 0.13
	MA (%)	80.72 ± 6.54	83.99 ± 1.62	80.97 ± 3.31	79.15 ± 3.29	100

RTCA—real time cell analyzer (xCELLigence system); MTT—3-[4,5-dimethylthiazole-2-yl]-2,5-diphenyltetrazolium bromide test; CI—cell index; A—adherence; OD—optical density; MA—metabolic activity. Significantly different compared to the control ** *p* < 0.01; ***p < 0.001.
